# Value of altered methylation patterns of genes RANBP3, LCP2 and GRAP2 in cfDNA in breast cancer diagnosis

**DOI:** 10.5937/jomb0-47507

**Published:** 2024-06-15

**Authors:** Qin Hu, Yu Mao, Haomiao Lan, Yi Wei, Yuehua Chen, Qiang Ye, Hongying Che

**Affiliations:** 1 Zigong Maternal and Child Health Hospital, Department of Clinical Laboratory, Zigong, China; 2 Zigong First People's Hospital, Department of Thyroid and Breast Surgery, Zigong, China

**Keywords:** methylation pattern, RANBP3, LCP2 GRAP2, cfDNA, breast cancer, obrazac metilacije, RANBP3, LCP2 GRAP2, cfDNA, karcinom dojke

## Abstract

**Background:**

The purpose of this study was to investigate the potential of plasma cfDNA methylation patterns in reflecting tumour methylation changes, focusing on three candidate sites, cg02469161, cg11528914, and cg20131654. These sites were selected for verification, with a particular emphasis on their association with breast cancer.

**Methods:**

We conducted a comprehensive analysis of 850k whole-methylation sequencing data to identify potential markers for breast cancer detection. Subsequently, we investigated the methylation status of the genes Ran-binding protein 3 (RANBP3), Lymphocyte cytoplasmic protein 2 (LCP2), and GRB2 related adaptor protein 2 (GRAP2), situated at the specified sites, using cancer and canceradjacent tissues from 17 breast cancer patients. We also examined the methylation patterns in different molecular subtypes and pathological grades of breast cancer. Additionally, we compared the methylation levels of these genes in plasma cfDNA to their performance in tissues.

**Results:**

Our analysis revealed that RANBP3, LCP2, and GRAP2 genes exhibited significant methylation differences between cancer and cancer-adjacent tissues. In breast cancer, these genes displayed diagnostic efficiencies of 91.0%, 90.6%, and 92.2%, respectively. Notably, RANBP3 showed a tendency towards lower methylation in HR+ breast cancer, and LCP2 methylation was correlated with tumour malignancy. Importantly, the methylation levels of these three genes in plasma cfDNA closely mirrored their tissue counterparts, with diagnostic efficiencies of 83.3%, 83.9%, and 77.6% for RANBP3, LCP2, and GRAP2, respectively.

**Conclusions:**

Our findings propose that the genes RANBP3, LCP2, and GRAP2, located at the identified methylation sites, hold significant potential as molecular markers in blood for the supplementary diagnosis of breast cancer. This study lays the groundwork for a more in-depth investigation into the changes in gene methylation patterns in circulating free DNA (cfDNA) for the early detection not only of breast cancer but also for various other types of cancer

## Abstract (sr)

**Uvod:**

Svrha ove studije je bila da se istraži potencijal obrazaca metilacije cfDNK plazme u odrazu promena metilacije tumora, fokusirajući se na tri kandidata, cg02469161, cg11528914 i cg20131654. Ove lokacije su odabrane za verifikaciju, sa posebnim naglaskom na njihovu povezanost sa rakom dojke.

**Metode:**

Sproveli smo sveobuhvatnu analizu 850.000 podataka sekvenciranja cele metilacije da bismo identifikovali potencijalne markere za otkrivanje raka dojke. Nakon toga, istražili smo status metilacije gena Ran-vezujući protein 3 (RANBP3), limfocitni citoplazmatski protein 2 (LCP2) i GRB2 povezan adapter protein 2 (GRAP2), koji se nalaze na navedenim mestima, koristeći kancer i susedna tkiva raka od 17 pacijenata obolelih od raka dojke. Takođe smo ispitali obrasce metilacije kod različitih molekularnih podtipova i patoloških stepena raka dojke. Pored toga, uporedili smo nivoe metilacije ovih gena u cfDNK plazme sa njihovim performansama u tkivima.

**Rezultati:**

Naša analiza je otkrila da geni RANBP3, LCP2 i GRAP2 pokazuju značajne razlike u metilaciji između raka i tkiva u blizini raka. Kod raka dojke, ovi geni su pokazali dijagnostičku efikasnost od 91,0%, 90,6% i 92,2%, respektivno. Značajno je da je RANBP3 pokazao tendenciju ka nižoj metilaciji kod HR+ raka dojke, a metilacija LCP2 bila je u korelaciji sa malignitetom tumora. Važno je da su nivoi metilacije ova tri gena u cfDNK u plazmi blisko odraz njihovih kolega u tkivu, sa dijagnostičkom efikasnošću od 83,3%, 83,9% i 77,6% za RANBP3, LCP2 i GRAP2, respektivno.

**Zaključak:**

Naši nalazi sugerišu da geni RANBP3, LCP2 i GRAP2, locirani na identifikovanim mestima metilacije, imaju značajan potencijal kao molekularni markeri u krvi za dodatnu dijagnozu raka dojke. Ova studija postavlja temelje za dublje istraživanje promena u obrascima metilacije gena u cirkulišućoj slobodnoj DNK (cfDNK) za rano otkrivanje ne samo raka dojke već i raznih drugih vrsta raka.

## Introduction

Breast cancer holds the top position in incidence among female cancers, constituting approximately 25% of all diagnosed cancers and contributing to 15% of cancer-related mortality [1] [2] [3]. In 2020 alone, China reported 416,371 new cases of breast cancer, leading to 117,174 deaths [4]. The complexity of breast cancer manifests in its heterogeneity, presenting multiple histological and molecular subtypes, diverse clinical behaviours, and variable treatment responses. Early detection plays a pivotal role in the successful treatment and prognosis of breast cancer. Subsequent postoperative follow-up efforts significantly influence the survival rate by monitoring potential recurrence [5] [6]. The challenges posed by late-onset symptoms, difficulties in evaluating tumour malignancy, and the unpredictable nature of the disease contribute to its elevated mortality rate [7]. Traditional breast cancer diagnostic methods encompass physical examination, molybdenum target X-ray examination, ultrasound imaging, magnetic resonance imaging, and tissue biopsy [8]. Breast cancer screening in China, initiated relatively late, primarily includes projects such as the »National Million Women Breast Cancer Survey Project« and »Two Cancers (Breast Cancer and Cervical Cancer) Screening.« Post-surgery follow-up relies on physical examination, ultrasound, and molybdenum target X-ray examination. However, molybdenum target X-ray examination has associated side effects. Additionally, small cancer foci close to or within the chest wall, as well as dense breasts, are prone to missed diagnoses [9] [10]. Detecting malignant lesions amidst the coexistence of benign and malignant lesions is challenging, and imaging pseudophase can lead to misleading results [11]. Physical and ultrasound examinations require substantial breast lesions for detection [12], highlighting the need for a more sensitive and specific method for early breast cancer recurrence detection.

Liquid biopsy, a hallmark of precision medicine, is increasingly utilized for early diagnosis, prognosis evaluation, recurrence assessment, and treatment monitoring [13]. Circulating cell-free DNA (cfDNA), primarily derived from apoptotic cells, is shown to be more than 90% tumour-derived [14]. With a half-life ranging from 16 minutes to 2.5 hours, cfDNA can provide real-time reflection of tumour load [15]. Circulating tumour DNA (ctDNA) in cfDNA is primarily employed to offer comprehensive information about the tumour genome, with gene mutations appearing specifically in tumour cells serving as tumour markers [16]. While tissue biopsy remains the clinical gold standard, it cannot overcome the heterogeneity of time and space. Small biopsy specimens may not accurately reflect the overall tumour situation and present other limitations [17] [18]. Studies indicate that liquid biopsy can track the evolutionary dynamics and heterogeneity of tumours, detecting early treatment resistance, residual disease, and recurrence [19]. Moreover, liquid biopsy, as a noninvasive sampling method, can be applied continuously at multiple time points to assist in monitoring disease progression [20]. Epigenetics encompasses heritable changes that do not alter the DNA sequence but significantly impact gene function. For instance, alterations in methylation patterns can enhance genomic instability, hindering the expression of tumour-suppressor genes [21] [22] [23]. Recent research has demonstrated a clear association between changes in DNA methylation patterns and the development of breast cancer [24] [25] [26]. Liquid biopsy relies on original tumour cells or DNA released into the blood, and cancer-related nucleic acid markers, including epigenetic changes, can also be released into the bloodstream. Consequently, the methylation patterns observed in primary breast tumours are akin to those found in the blood [19] [27].

Notably, Xu et al.’s [28] study revealed that even before tumours are clinically detected, DNA methylation spectra in the blood start to undergo changes indicative of invasive breast cancer. While DNA methylation analysis is a rapidly evolving field, the development of repeatable epigenetic blood tests for breast cancer diagnosis and follow-up has not yet advanced into routine clinical tests. The objective of this study was to identify highly sensitive and specific plasma-free methylated genes, aiming to validate the efficacy of these novel biomarkers in breast cancer diagnosis and follow-up.

## Materials and methods

### Subjects and study design

The trial received approval from the review committee of the Maternal and Child Health Care Hospital of Zigong, and all participating patients provided written informed consent. Inclusion criteria involved patients with confirmed breast cancer through biopsy or postoperative pathology. From October 2020 to May 2021, a total of 17 women underwent screening as part of standard preoperative evaluations. During surgery, samples of cancer tissue, cancer- adjacent tissue, and peripheral blood were obtained. Tumour evaluations were conducted by the pathology department.

Out of the 45 samples, 29 were collected prior to treatment, 10 were obtained at least three months after surgery with the patients being disease-free, and 6 were collected during a recurrent state post-surgery. Methylation sequencing of breast cancer tissue and blood was performed using the 850K chip. Candidate genes and sites were analysed, and the three genes selected in this study underwent verification.

### Methods

#### DNA extraction from cancer tissues, cancer-adjacent tissues, and plasma

Genomic DNA of cancer and cancer-adjacent tissues was extracted using the QIAamp DNA FFPE tissue kit (Qiagen, Hilden, Germany), and cfDNA was extracted using the CapitalBio Genomiccs nucleic acid extraction and purification kit. Nanodrop 2000 detect the concentration must be ≥20 ng/μL and total DNA ≥ 400 ng. Purity: OD260/280 =1.7∼1.9; OD260/230 ≥ 2.0.

#### DNA methylation experiments

Every 500 ng of DNA was treated with sodium bisulfite using an EZ DNA methylation kit (Zymo Research Company, USA). The product was subjected to target fragment multiplex PCR amplification. According to the candidate site design primer for amplification, the primer sequence for cg02469161 used was F’-TGATAGGTTGTTTTAGTTGTTGT, R’- ATAAAAAACRTACCTACCCTACT, and the amplification product was 103 bp; the primer sequence for cg11528914 used was F’-GATTATATTTGTTYGTAGAGGAAG, R’-AACRTTCTTAACTCTTCCAACT, and the amplification product was 120 bp; the primer sequence for cg20131654 used was F’-GTGGGTTTAGGGTTTTATATTTT, R’-CTTCAAATATTCAAACTAAACRCCT and the amplification product was 116 bp. High-throughput sequencing was performed using the Illumina HiSeq platform in 2×150 bp paired-end sequencing mode to obtain FastQ data.

#### Statistical analysis and mapping

The data were mainly analysed using R software and the ChAMP software package. The DNA methylation level conformed to a normal distribution. A t test was used to compare the DNA methylation level of breast cancer tissue with that of cancer-adjacent tissue and preoperative and postoperative patient blood. One-way ANOVA or the Mann Whitney U test was used for multiple comparisons. P<0.05 indicates statistical significance. Med-Calc 15.2.2 (Med-Calc, Mariakerke, Belgium) was used to draw a receiver operating characteristic (ROC) curves and calculate the area under the curve (AUC). Origin9 was used to draw box plots. It is worth noting that in the case of multiple comparisons, we took appropriate statistical measures to adjust the P value to ensure the reliability of the results. In addition, we considered potential confounders and adjusted accordingly in the analysis to increase the rigor and credibility of the study.

## Results

### Molecular biomarkers of DNA methylation in breast cancer

We performed 850k chip whole methylation sequencing on breast cancer tissue, cancer-adjacent tissue and cfDNA of breast cancer patients, analysed differential sites and regions, and screened candidate sites and genes.

**Figure 1:**
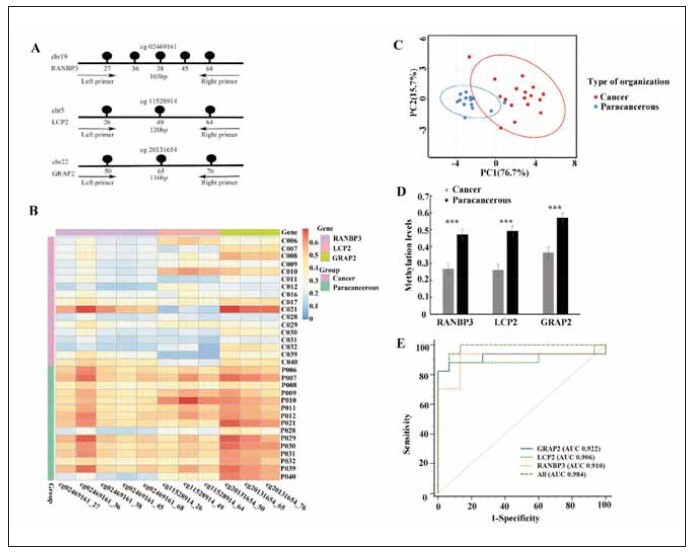
Markers of breast cancer tissue. (A) Candidate sites were screened out using 850k chip data and then verified. A schematic of the targeted amplification approach for each targeted region. The names of the genes where a CpG is located is indicated. Each lollipop represents a CpG detection site. The length of the amplicon in nucleotide base pairs (bp) appears below it. (B) Methylation heatmaps of cancer and paracancerous tissue target genes from 17 breast cancer patients. Two cases of paracancerous tissue were not included due to unqualified sampling. (C) Principal component analysis (PCA) showed significant differences in methylation levels of the RANBP3, LCP2 and GRAP2 genes between cancer and paracancerous tissues. (D) Methylation levels of RANBP3, LCP2 and GRAP2 in cancer and paracancerous tissues were compared in a bar chart (independent- sample t test). ***, P<0.005. (E) ROC curve for diagnosing breast cancer. Breast cancer methylation level values were defined using the mean of all three markers (All) or the mean of the three markers independently.

To verify that the sites cg02469161, cg11528914 and cg20131654 have different methylation patterns in cancer and cancer-adjacent tissues corresponding primers were designed for amplification of the target region. The target amplification method for each target region is illustrated in Figure 1A. Each lollipop represents a CpG detection site, and the corresponding number is the location of the methylated base. The name of the gene where the detected fragment is located is marked, and the genes in the methylation region are RANBP3, LCP2 and GRAP2. The amplified target nucleotide base pair length (bp) is shown below it. Heat maps were used to show methylation levels in cancer and paracancer tissues of 17 breast cancer patients (Figure 1B). The blue to red color transition represents hypomethylation to hypermethylation. It can be seen that cancer tissues tend to be hypomethylation and paracancer tissues tend to be hypermethylation. Principal component analysis (PCA) showed significant differences in the methylation levels of target genes in breast cancer and adjacent tissues (Figure 1C). The RANBP3, LCP2 and GRAP2 genes showed low methylation levels (0.268±0.048, 0.263±0.029, 0.364±0.020) in cancer tissue and high levels (0.472±0.065, 0.491± 0.036, 0.569±0.042) in cancer-adjacent tissue, with significant differences (*P*<0.005 *P*<0.005 *P*<0.005) (Figure 1D). A ROC curve showed that when the sensitivity was 86.67% (RANBP3), 82.35% (LCP2) and 88.24% (GRAP2), the specificity of breast cancer diagnosis was 93.3%; the sensitivity of identifying breast cancer patients by the comprehensive methylation level of the three genes was 94.1%, with a specificity of 93.3%. Using all three DNA methylation markers had higher sensitivity and an area under the curve (AUC 0.984) than using any single marker alone (Figure 1E).

### RANBP3, LCP2 and GRAP2 gene methylation levels are related to molecular subtype and tumour malignancy in breast cancer

To analyse the influencing factors of the three-gene methylation pattern in breast cancer patients, we collected various clinical pathological features. The methylation level of the RANBP3 gene was found to be related to the molecular subtype of breast cancer (P=0.001, one-way analysis of variance), and the methylation level of the LCP2 gene was associated with the malignancy degree of tumours (P=0.011, one-way analysis of variance), as indicated in Table 1.

**Table 1:** Relationships between clinical characteristics and gene methylation levels. P values <0.05 are in bold. Ki-67, marker of proliferation. a, Independent sample t test. b, One-way analysis of variance

Variable	Number	RANBP3 <br>Methylation <br>Level	P value	LCP2 <br>Methylation <br>Level	P value	GRAP2 <br>Methylation <br>Level	P value
Age^a^							
≥50	13	0.282	0.497	0.26	0.51	0.372	0.868
<50	4	0.248		0.295		0.395	
Tumor size^b^							
T1	3	0.199	0.392	0.192	0.386	0.326	0.765
T2		14	0.287		0.285		0.377
Lymph node metastasis^b^							
N0	12	0.269	0.844	0.240	0.127	0.75	0.360
N1		4	0.297		0.313		0.409
N2		1	0.229		0.488		0.312
AJCC clinical stage^a^							
I∼II A/B	16	0.268	0.427	0.262	0.452	0.372	0.794
III∼IV		1	0.311		0.329		0.399
Pathology grade^b^							
1	1	0.229	0.554	0.488	0.011	0.312	0.852
2		9	0.252		0.313		0.361
3		7	0.308		0.177		0.387
Molecular subtypes^b^							
HR HER2	10	0.229	0.001	0.314	0.25	0.339	0.116
HR HER2	4	0.264		0.239		0.364	
HR- HER2+		2	0.378		0.156		0.389
HR- HER2-		1	0.579		0.136		0.662
Ki67^a^							
<30%	6	0.281	0.782	0.311	0.262	0.331	0.302
≥30%	11	0.267		0.243		0.392	
Vascular invasion^a^							
NO	16	0.281	0.193	0.267	0.766	0.377	0.223
YES	1	0.134		0.308		0.211	
Neural invasion^a^							
NO	16	0.275	0.688	0.257	0.078	0.372	0.671
YES	1	0.2299		0.488		0.312	

**Figure 2:**
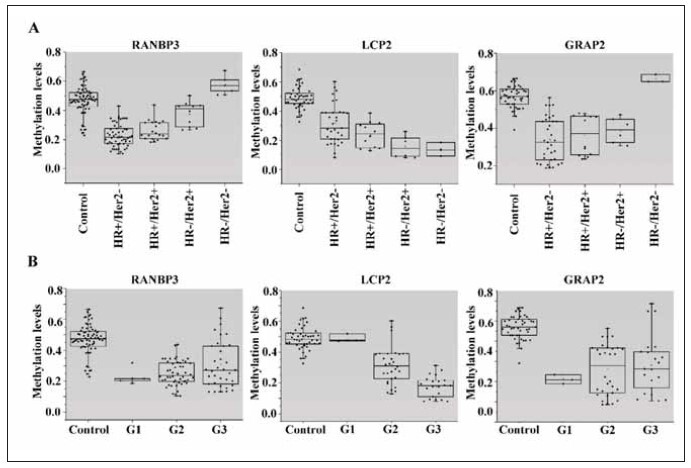
Analysis of the relationship between molecular subtype, grade of pathology and three genes. (A) Methylation levels in breast cancer and paracancer are shown above, with samples separated by receptor status at initial biopsy. Methylation levels of RANBP3, LCP2 and GRAP2 in the breast cancer group correlated with tumour molecular characteristics (P< 0.05, Tukey HSD). (B) Samples were separated by pathological grade at the time of initial biopsy. Methylation levels of RANBP3, LCP2 and GRAP2 in the breast cancer group correlated with tumour malignancy (P <0.05, Tukey HSD).

Subsequently, we conducted a more in-depth analysis of the three-gene methylation pattern between cancer-adjacent tissues and different molecular subtypes and pathological grades of breast cancer. The methylation level of the RANBP3 gene in HR+/HER2-, HR+/HER2+, and HR-/HER2+ types significantly differed between cancer-adjacent tissues and cancer tissues (P=0.000, P=0.000, P=0.013, Tukey HSD). However, no significant difference was observed in HR-/HER2- type breast cancer (P=0.06, Figure 2A). Due to the small sample size in this group, it may not reflect the real situation, and we plan to expand the sample size for further study if conditions permit. Interestingly, the methylation level of the RANBP3 gene in cancer tissues of the HR+/HER2- and HR+/HER2+ types was significantly lower than that in the HR-/HER2+ type (P=0.000, P=0.007), suggesting a correlation between the methylation level of the RANBP3 gene and HR expression in breast cancer. Similarly, significant differences were found in GRAP2 gene methylation levels between cancer-adjacent tissues and cancer tissues among different molecular subtypes (P=0.000, P=0.000, P=0.000, Tukey HSD). However, there was no significant difference in HR- /HER2+ breast cancer (P=0.408, Figure 2A). Moreover, there was no significant difference in GRAP2 gene methylation levels among the HR+/HER2-, HR+/HER2+, and HR-/HER2+ molecular subtypes. The overall methylation level of the LCP2 gene in cancer-adjacent tissues was significantly higher than that in cancer tissues of various subtypes (P<0.005, Figure 2A), and there was no significant difference in methylation levels within molecular subtype groups.

Considering the tumour malignancy degree, we further analysed the correlation between the three-gene methylation patterns and tumour malignancy degree. Similar to RANBP3 and GRAP2 genes mentioned above, we observed significant differences in average methylation levels between adjacent tissues and various grades of cancer tissues (P<0.05, Tukey HSD), but with no relationship to malignancy degree. The methylation level of the LCP2 gene decreased with increasing tumour malignancy (P<0.05, Figure 2B). Therefore, it can be concluded that the methylation level of the RANBP3 gene is related to HR expression in breast cancer, and the methylation level of the LCP2 gene is associated with the degree of tumour malignancy.

### Methylation levels of three genes in cfDNA from breast cancer patients changed

**Figure 3:**
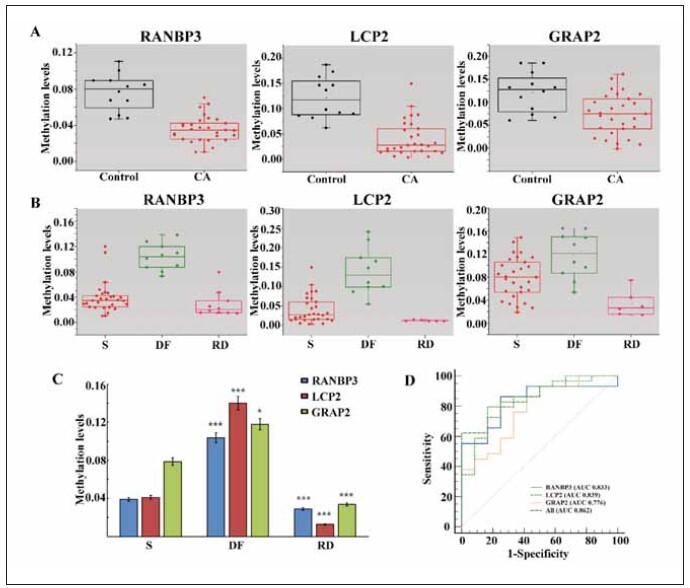
Methylation levels of three genes changed in the blood of breast cancer patients. (A) Plasma methylation levels of three breast markers were compared between healthy controls (n =12) and breast cancer patients (n =29). Methylation levels of three breast markers were significantly reduced in the cancer patients (t test). (B) Methylation levels of three kinds of breast marker genes in the plasma of patients at initial diagnosis (n =29), disease free after surgery (n =10) and with recurrence after surgery (n =6) were compared. At least three months after surgery, methylation levels of three breast markers were significantly increased (t test) and decreased when recurrence occurred. (S: at initial diagnosis; DF: disease free after surgery; RD: recurrent disease after surgery). (C) Methylation levels of three kinds of breast markers in the plasma of patients at initial diagnosis (n =29), disease free after surgery (n =10) and with recurrence after surgery (n =6) are compared in a bar chart. (*, P<0.05; ***, P<0.005). (D) ROC curve of the cfDNA methylation level for diagnosis of breast cancer. The comprehensive diagnostic level of blood was determined using the average level of the three markers.

Moreover, to investigate whether cfDNA exhibits a methylation pattern in the three genes similar to that of tissue, we included 12 healthy women and 29 newly diagnosed breast cancer patients. Methylation levels of the three genes in cfDNA of cancer patients were lower than those in the control group (P<0.05, Figure 3A), consistent with the tissue methylation pattern. At least three months after breast cancer surgery, methylation levels of the three genes in cfDNA increased compared to the initial diagnosis (P<0.05) and decreased after breast cancer recurrence (P<0.05) (Figure 3B, Figure 3C). This indicates that the occurrence of breast cancer is associated with the low methylation status of these three genes.

The sensitivity and specificity of cfDNA methylation levels for diagnosing breast cancer are depicted in Figure 3D. The diagnostic efficiency of RANBP2, LCP2, and GRAP2 gene methylation levels was 83.3%, 83.9%, and 77.6%, respectively, and the combined diagnostic efficiency was 86.2%.This indicates that changes in the methylation pattern of these three genes are closely related to breast cancer development. Based on this, we consider whether the methylation levels of these three genes can be used as blood markers for screening breast cancer. In the follow-up study, we will make a baseline of the blood methylation levels of RANBP3, LCP2, and GRAP2 genes in healthy people. When the methylation levels of RANBP3 are reduced, we will be alert to the occurrence of HR+ breast cancer. When the methylation level of LCP2 gene is reduced, the greater the degree of reduction, the more malignant degree of cancer may occur. Combined pathological grading provides reference for breast cancer surgery, and combined three-gene diagnosis is more efficient, which can provide help for breast cancer screening and follow-up monitoring after breast cancer surgery.

## Discussion

In this study, we utilized 850k chip methylation sequencing to analyse the methylation patterns of breast cancer patient cancer and para-cancerous tissues at cg02469161, cg11528914, and cg20131654 sites, revealing distinct methylation patterns. Furthermore, we conducted a verification of the accuracy of these differences for the three genes located at these sites. While some previous studies have explored the relationship between the methylation of specific genes and breast cancer [6] [29], our study stands out as the first to comparatively examine peri pheral blood cfDNA methylation patterns and breast cancer tissue methylation patterns. Additionally, it is the initial investigation to validate the association between RANBP2, LCP2, and GRAP2 gene methylation and breast cancer.

Our findings demonstrated that the methylation levels of all three genes were linked to the occurrence of breast cancer. Specifically, the methylation level of the RANBP3 gene exhibited a more pronounced tendency to change in estrogen receptor-expressing breast cancer, while the methylation level of the LCP2 gene was associated with tumour malignancy. Moreover, cfDNA from breast cancer patients exhibited a methylation pattern of these three genes that mirrored that of cancer tissue. The RAN-binding protein 3 (RANBP3) gene, situated on chromosome 19, has been predominantly associated with the proliferation of leukaemia cells [30] and male sperm production [31], with limited studies exploring its connection to breast cancer. Notably, the anthracycline drug doxorubicin (Dox), a primary treatment for breast tumours and adjuvant therapy drug, induces significant toxicity to the myocardium while eliminating cancer cells. Mesenchymal stem cells, known to secrete a substantial amount of RANBP3 protein, play a role in negatively regulating cell proliferation through the TGF-b signalling pathway, thus mitigating Dox-induced cell toxicity [32].

Our study revealed that the RANBP3 gene exists in a low methylation state in breast cancer tissue and is correlated with hormone receptor (HR) expression. Considering the characteristic of unchecked proliferation in cancer cells and without accounting for the influence of transcription and protein expression factors, we postulate that the RANBP3 gene may be highly expressed in breast cancer due to some demethylation mechanism, thereby promoting cancer cell proliferation. However, further research is necessary to substantiate this hypothesis. Specifically, the observed lower methylation level of the RANBP3 gene in HR+ breast cancer compared to HR- breast cancer prompts an exploration of the relationship between RANBP3 gene methylation levels and triplenegative breast cancer. The lymphocyte cytosolic protein 2 (LCP2) gene, situated on chromosome 5, encodes a substrate of the tyrosine kinase pathway of the T-cell antigen receptor (TCR) activation protein. LCP2 plays a crucial role in TCR-mediated intracellular signal transduction. Prior studies have identified LCP2’s association with breast cancer, and genetic variations in LCP2 have been linked to postoperative secondary lymphedema in breast cancer. Our research builds on these findings, demonstrating that the methylation level of the LCP2 gene correlates with the degree of malignancy in breast cancer. Specifically, higher pathological malignancy corresponds to lower methylation levels. Additionally, our study holds the advantage of confirming that the methylation pattern of LCP2 in the peripheral blood of cancer patients mirrors that in cancer tissue, allowing for convenient and real-time monitoring of tumour development. The methylation level of the LCP2 gene increases in breast cancer patients who experience recurrence after surgery, displaying a significant difference compared to patients without recurrence. Thus, changes in LCP2 gene methylation serve as a potential marker for cancer occurrence and offer diagnostic efficiency of 83.9% [33] [34] [35] [36].

GRAP2, located on chromosome 22q13.1, encodes a receptor-like protein associated with the leukocyte-specific protein tyrosine kinase signalling pathway. Previous studies have implicated GRAP2 in tumorigenesis, with interactions enhancing Cyclin D1 expression. Our research, however, indicates that while GRAP2 is highly expressed in nontriple-negative breast cancer, it exhibits no relation to methylation in triple-negative breast cancer. This discrepancy may be attributed to a small sample size or may warrant further exploration in larger cohorts for validation. The diagnostic efficiency of changes in the methylation pattern of the GRAP2 gene in tissue was the highest among the three genes, reaching 92.2%, yet its efficiency in cfDNA was the lowest at 77.6%. The suitability of changes in GRAP2 gene methylation levels as blood diagnostic markers for breast cancer requires further discussion [37] [38] [39] [40].

Despite our efforts to design the study to ensure its internal and external validity, there are several limitations that need to be considered. First, due to the observational nature of the study, we cannot rule out potential observational bias. Although we made appropriate statistical adjustments to control for confounding factors, we cannot completely exclude the possible influence of other unmeasured factors on the study results. Second, the selection of the sample may be subject to selection bias, so our results may not be applicable to the entire population. We encourage future studies to replicate our findings in larger samples to validate our findings. Additionally, the specific population characteristics used in the study may limit the extrapolability of the results, so caution is needed when generalizing the results to other populations. Third, we acknowledge that our study may be affected by other potential confounders, such as lifestyle, genetic factors, etc., which may have an impact on the study results. Although we included some controls in our analyses, the potential influence of other unknown factors cannot be ruled out. Finally, our findings only represent observations at a specific point in time and cannot capture changes over time. To gain a more complete understanding of the development of the phenomenon, longer follow-up studies are needed.

In conclusion, our study highlights alterations in the methylation patterns of RANBP2, LCP2, and GRAP2 genes in breast cancer tissue, aligning with consistent changes in methylation levels in blood cfDNA. These changes serve as promising blood molecular markers for the auxiliary diagnosis of breast cancer. While acknowledging study limitations, such as a relatively small sample size for triple-negative breast cancers and the need for further exploration of transcription and protein levels, our findings present valuable insights into the potential diagnostic and prognostic implications of gene methylation in breast cancer. Future research should delve into the biological significance of these methylation sites, unravel the underlying mechanisms, and explore potential pathways influencing breast cancer occurrence and progression.

## Dodatak

### Funding

This work was supported by the key project of the Zigong Science and Technology Bureau (2020ZC29).

### Data Availability Statement

All data generated or analysed during this study are included in this published article.

### Conflict of interest statement

All the authors declare that they have no conflict of interest in this work.
